# Vitamin D Binding Protein Affects the Correlation of 25(OH)D and Frailty in the Older Men

**DOI:** 10.1155/2014/543783

**Published:** 2014-04-13

**Authors:** Yi Wang, Yan-Jiao Wang, Jun-Kun Zhan, Zhi-Yong Tang, Wu Huang, Pan Tan, Shan Gao, Cai-Li Ma, Zai-Jin Jian, You-Shuo Liu

**Affiliations:** Geriatric Department of the Second Xiang-Ya Hospital, Institute of Aging and Geriatric, Central South University, Changsha, Hunan 410011, China

## Abstract

Vitamin D binding protein (DBP) may alter the biologic activity of 25-hydroxyvitamin D [25(OH)D]. The objective of our present study was to determine the joint effect of serum 25(OH)D and DBP on the risk of frailty. Five hundred sixteen male participants aged 70 years or older were recruited in Changsha city and its surrounding area in Hunan province of China. Frailty was defined as the presence of at least three of the five following criteria: weakness, low physical activity, slow walking speed, exhaustion, and weight loss. Multivariate linear regression analysis was performed to assess the relationship between 25(OH)D and DBP levels. Odds ratios (ORs) for frailty were evaluated across quartiles of 25(OH)D and DBP levels, adjusted age, education, and body mass index. The results showed that participants in the lowest quartile of 25(OH)D and the highest quartile of DBP levels, the lowest quartile of 25(OH)D and the lowest quartile of DBP levels, and those in the the lower quartile of 25(OH)D and lowest quartile of DBP levels had significantly higher OR of being frail compared with those in the highest quartile of 25(OH)D and lowest quartile of DBP, with OR of 3.18 (95% CI: 1.46–4.56, *P* < 0.05), 2.63 (95% CI: 1.31–3.68, *P* < 0.01), and 2.52 (95% CI: 1.22–3.52, *P* < 0.05), respectively. The results indicate that the joint effect of serum 25(OH)D and DBP levels is associated with the risk of frailty, and serum DBP levels affects 25(OH)D-frailty relationship in the older men.

## 1. Introduction

In the elderly, frailty is an extremely common clinical state and a serious health problem in which there is an increase in an individual's vulnerability for developing increased dependency and/or mortality when exposed to a stressor [1, 2]. Overall, it appears that low levels of vitamin D play an important role in frailty. Vitamin D insufficiency may exacerbate frailty by affecting mainly two aspects, bone formation and neuromuscular function [3, 4]. Vitamin D is expected to be a treatment for frailty in an aging society [5, 6].

Studies examining the relationship between total circulating 25-hydroxyvitamin D [25(OH)D] levels and frailty have yielded mixed results. Many epidemiologic investigations have suggested that lower levels of 25(OH)D have been linked to muscle strength and increased risk of frailty [7–15]. Additionally, vitamin D supplementation reduces falls and improves muscle function in people with low 25(OH)D levels [16–18]. However, not all observational studies have confirmed the relationship between 25(OH)D and the risk of frailty. In several randomized trials, no effect of vitamin D supplementation on the risk of frailty was observed [19, 20]. It is possible that, in these studies by using total 25(OH)D levels as an only measure of vitamin D status, individuals may be misclassified as sufficient or insufficient in vitamin D.

The free hormone hypothesis postulates that only hormones liberated from binding proteins enter cells and produce biologic action [21]. 25(OH)D circulates bound to vitamin D binding protein (DBP) (85% to 90%) and albumin (10% to 15%), with less than 1% of circulating hormone in its free form [22]. DBP may alter the biologic activity of 25(OH)D. However, the link between the biologic activity of 25(OH)D and frailty is not clear yet. We hypothesized that the joint effect of serum 25(OH)D and DBP levels is tightly linked to the risk of frailty and serum DBP levels might affect the correlation of 25(OH)D and frailty.

## 2. Material and Methods

### 2.1. Participants

From November 2012 to March 2013, 1048 aged men who were ≥70 years old were recruited in Changsha city and its surrounding area in Hunan province of China. Individuals were originally excluded if they were unable to walk without the assistance of another person or their renal function and liver function were being abnormal. Five hundred sixteen subjects had sufficient blood samples for analysis, and their characteristics are presented in Table 1. The study protocol was approved by the Second Xiangya Hospital of Central South University Ethics Committees in accordance with the Declaration of Helsinki and Good Clinical Practices Guidelines.

### 2.2. Assessment Methods

#### 2.2.1. Biochemical Analysis

Fasting morning blood was collected and serum was divided into aliquots and they were stored at −70°C until they were shipped on dry ice to a central laboratory, where they were stored at −70°C until analysis. Analysis was performed using the radioimmune assay kit (DiaSorin, Stillwater, MN, USA) to measure 25(OH)D. Intra- and interassay coefficients of variation (CVs) for 25(OH)D were 6.3% and 9.1%, respectively. DBP was measured by commercial enzyme-linked immunosorbent assay (ELISA; R&D Systems, Minneapolis, MN, USA) according to the manufacturer's instructions. The assay was conducted after diluting serum with intra- and interassay CV 6.1% and 10.2%, respectively, for DBP. The manufacturer reports that DBP has no significant cross-reactivity with human albumin, vitamin D_3_, or a-fetoprotein. Intact PTH was measured by electrochemiluminescence immunoassay on the Cobas E160 automated analyzer (Roche Diagnostics, Indianapolis, IN, USA). The intra- and interassay CVs for intact PTH measurement were 4.7% and 9.6%, respectively. Calcium and albumin levels were measured by dye-based photometric assays on an automated analyzer.

#### 2.2.2. Calculation of Free 25(OH)D

Free levels of 25(OH)D were calculated using the following equation [23]:

(1) Free 25(OH)D  =total  25(OH)D1+(6×103×albumin)+(7×108×DBP).

The reported correlation coefficient between calculated free 25(OH)D using this equation and measured free 25(OH)D by centrifugal ultrafiltration is 0.925 [23].

#### 2.2.3. Other Measurements

Participants completed a questionnaire and were interviewed at the examinations and asked about health status, educational achievement, and smoking status. A selected medical history including a history of a physician diagnosis of stroke, cancer, dementia, hypertension, Parkinsonism, diabetes mellitus, coronary heart disease, and chronic obstructive lung disease was obtained. Participants were asked to bring all medications including nonprescription supplements to clinic for verification of use. Body weight and height measurements were used to calculate a standard body mass index (BMI).

#### 2.2.4. Frailty Status

Participants were classified as frail, prefrail, and nonfrail according to a validated screening tool based on the presence or absence of five measurable characteristics by Fried and Walston [2]: weakness, low physical activity, slow walking speed, exhaustion, and weight loss. “Weakness” was defined as grip strength in the lowest quintile within groups defined by sex and BMI. Participants reported their level of daily leisure physical activity in the past year; “low physical activity” was defined as either complete inactivity or performing low-intensity activities less than 1 h/wk. “Slow walking speed” was defined as usual walking speed in the slowest quintile within groups defined by sex and height. Walking speed was measured on a 4-m course using photocell recordings at the course start and finish. The final measure averaged two walks. “Exhaustion” was indicated by a response of “occasionally” or “often/always” to the statement, “I felt that everything was an effort.” “Weight loss” was measured as self-reported unintentional weight loss more than 4.5 kg within the past year. Individuals with a critical mass of 3 or more of the five components were defined as frail, those with one or two components were defined as prefrail, and those with none were defined as nonfrail.

### 2.3. Statistical Analysis

Quartile cutpoints for DBP and 25(OH)D were determined based on the distribution among participation. Summary statistics were constructed for comparing baseline characteristics of the participation. Distributions of sociodemographic and health characteristics, total 25(OH)D, and free 25(OH)D and DBP levels were summarized according to frailty status. The Spearman rank correlation coefficient was used to describe the correlation between 25(OH)D and DBP levels. Linear regression analysis was used to study the relationship between total 25(OH)D (as independent variable) and DBP levels (as dependent variable), adjusting for age, education, BMI, and smoking status. Logistic regression models were used to assess the effects of 25(OH)D and DBP levels on the risk of being frail versus nonfrail cross-sectionally at baseline. Because exploratory analyses suggested potential nonlinear associations of 25(OH)D and DBP levels with frailty, 25(OH)D and DBP levels were modeled as quartiles in association with frailty for ease of interpretation. Interaction terms were added to the main effects model to explore potential synergy between 25(OH)D and DBP levels in their associations with frailty.

## 3. Results

Characteristics of the subjects by quartiles of serum DBP are shown in Table 1. There was no significant difference in age, education, BMI, physical activity during leisure time, season of blood draw, vitamin D, and calcium supplementation among the four groups. 25(OH)D and DBP levels were correlated (Spearman correlation coefficient = 0.16, *P* < 0.05) adjusting for age, education, and BMI. Calculated free 25(OH)D levels were positively correlated with total 25(OH)D levels (*r* = 0.24, *P* < 0.05).

Table 2 reports baseline demographic and health-related characteristics, 25(OH)D, and DBP and free 25(OH)D levels of the study sample across frailty categories. There were significant differences in mean 25(OH)D and DBP levels across frailty categories (*P* < 0.05 for stepwise increase or decrease trend). Compared with nonfrail participants, frail participants were older (*P* < 0.05) and were less educated (*P* < 0.05).

To investigate potential joint association of 25(OH)D and DBP levels with frailty, odds ratios (ORs) of participants being frail versus nonfrail were assessed across quartile of 25(OH)D and DBP levels. As shown in Table 3, participants in the lowest quartile of 25(OH)D (Q1a) and the highest quartile of DBP (Q4b) levels, those in the lowest quartile of 25(OH)D (Q1a) and the lowest quartile of DBP (Q1b), and those in the lowest quartile of DBP (Q1b) and the lower quartile of 25(OH)D (Q2a) levels had significantly higher OR of being frail compared with those in the highest quartile of 25(OH)D (Q4a) and lowest quartile of DBP (Q1b) (reference group), with OR of 3.18 (95% CI: 1.46–4.56, *P* < 0.05), 2.63 (95% CI: 1.31–3.68, *P* < 0.01), and 2.52 (95% CI: 1.22–3.52, *P* < 0.05), respectively, adjusting for age, BMI, and education. These results showed that, in the setting of the lowest quartile of 25(OH)D levels, both the lowest and the highest DBP levels confer increased risk for frailty, suggesting a “ U-” shaped joint association of 25(OH)D and DBP levels with frailty, and that, in the setting of the lowest quartile of DBP levels, both the lowest and lower 25(OH)D levels confer increased risk for frailty. The interaction terms between quartile of 25(OH)D and DBP, however, were not statistically significant.

## 4. Discussion

This study has observed multiplicative interaction in the associations of DBP and 25(OH)D levels with frailty. The results suggest an association of increased levels of DBP and decreased levels of 25(OH)D with frailty (Table 3). Therefore, a high level of DBP or low level of 25(OH)D may increase the risk of frailty, whereas a low level of DBP or high level of 25(OH)D may reduce the risk of frailty.

Vitamin D status is determined by vitamin D stores in vivo and its biologic activity. Circulating 25(OH)D levels generally are considered to better reflect overall vitamin D stores [24]. The free hormone hypothesis postulates that only hormones liberated from binding proteins enter cells and produce biologic action [21]. 25(OH)D circulates binds to vitamin D binding protein (DBP) (85% to 90%) and albumin (10% to 15%), with less than 1% of circulating hormone in its free form [22]. In the present study, we have found that calculated free 25(OH)D levels were positively correlated with total 25(OH)D levels. Consistent with the free hormone hypothesis, the results of our study suggest that circulating DBP is an inhibitor of the biologic action of vitamin D in frailty patients. Unlike binding to DBP, binding to albumin does not inhibit the action of 25(OH)D. DBP behaves similarly to other serum hormone carrier proteins and has broad clinical applications. Like thyroid hormone-binding globulin and sex hormone-binding globulin, DBP may act as a serum carrier and reservoir, prolonging the circulating half-life of vitamin D while at the same time regulating its immediate bioavailability to target tissues [21]. Thus hormonal activity and sufficiency may be reflected by the amounts of bioavailable vitamin, not by total levels. So, a low DBP level may be beneficial due to the higher level of bioavailable 25(OH)D but may also be a bad thing considering vitamin D effects in tissues that express megalin. Indeed, as DBP is internalized in some cells through a megalin cubilin uptake, a higher DBP concentration may be a favourable point for these effects [25]. The affinity of DBP for 25(OH)D depends on the DBP genotype with important consequences on the calculation of free or bioavailable 25(OH)D [25]. A recent paper by Powe et al. [26] reports that community-dwelling black Americans had low levels of total 25(OH)D and DBP, resulting in similar concentrations of estimated bioavailable 25(OH)D as compared with whites. Racial differences in the prevalence of common genetic polymorphisms provide a likely explanation for this observation.

Currently, clinical testing for vitamin D deficiency is based on measurement of total serum concentrations of 25(OH)D [24]. Our data suggest that concentrations of total serum 25(OH)D may not be the best measure of assessing vitamin D insufficiency or sufficiency status in frailty patients. For example, aged men with high levels of DBP may appear to be 25(OH)D-sufficient but actually there may be higher risk of frailty due to deficient in bioavailable 25(OH)D. Joint examination of serum 25(OH)D and DBP concentrations could be better to reflect overall vitamin D stores and the biologic action of vitamin D.

Clinical trials of vitamin D supplementation in older frailty patients with low vitamin D status mostly report improvements in muscle performance and reductions in falls [16–18]. The underlying mechanisms are probably both indirect via calcium and phosphate and direct via activation of the vitamin D receptor (VDR) on muscle cells and bone by 1,25-dihydroxyvitamin D [1,25(OH)_2_D_3_]. VDR activation at the genomic level regulates transcription of genes involved in calcium handling and muscle cell or osteoblast differentiation and proliferation [3, 4, 27, 28].

This study has three limitations. First, sample size of the subgroups in the analysis across quartiles of DBP and 25(OH)D levels is relatively small and provides limited statistical power. Cautious interpretation of these results is warranted, and further investigation of the joint effects of DBP and 25(OH)D levels on frailty is needed. Second, genotyping of these subjects was not done. Third, other endocrine factors including IGF-1 and testosterone have been identified for their interactions with DBP and 25(OH)D as well as their associations with frailty [29]. Therefore, findings from this study should be interpreted in the context of the complexity of the nutrition and endocrine systems as well as multifactorial nature of the frailty syndrome. Despite these limitations, results from this study do support the free hormone hypothesis and provide a basis for further investigations into optimal vitamin D status in frailty individuals.

## 5. Conclusion

We conclude that the joint effect of serum 25(OH)D and DBP levels may be tightly linked to frailty, or serum DBP levels modify 25(OH)D-frailty relationship in the older men. Our findings support the hypothesis that the biologic activity of 25(OH)D may be altered by DBP in frailty patients and suggest that joint examination of serum 25(OH)D and DBP concentrations in future studies could shed additional light on the role of vitamin D and its pathway cofactors in the aetiology of frailty.

## Figures and Tables

**Table 1:** Elected baseline characteristics across quartiles of vitamin D binding protein (medians or proportions).

Characteristic	Quartile of serum vitamin D binding protein (nmol/L)				*P* value
	Q1 <4046	Q2 4046–<5269	Q3 5269–<6686	Q4 ≥6686	
Age (years)	76.5	75.1	76.8	77.3	Matched
Height (cm)	170	169	171	172	0.89
Weight (kg)	74.5	78.2	76.1	77.2	0.43
BMI (kg/m^2^)	24.6	25.1	24.2	25.5	0.61
Education (% > elementary)	16.0	14.6	14.1	13.8	0.49
Physically active during leisure time (% yes)	18.5	16.9	22.1	23.3	0.21
Season of blood draw (% Nov.–Mar.)	100	100	100	100	Matched
Supplemental vitamin D use (% yes)	28.1	26.7	15.3	14.6	0.16
Supplemental calcium use (% yes)	29.7	30.0	23.3	20.1	0.23
Serum biomarkers					
25(OH)D (nmol/L)	46.1	42.5	41.2	45.6	0.27
DBP (nmol/L)	3642	4632	5861	7183	NA
Free 25(OH)D (pmol/L)	26.5	28.4	23.9	22.2	0.13
Albumin (g/L) (normal range: 40–55 g/L)	36.0	37.7	38.3	39.1	0.36
Serum calcium (mmol/L) (normal range: 2.03–2.54 mmol/L)	2.1	2.2	2.1	2.3	0.46
Parathyroid hormone (ng/L) (normal range: 20.3–46.8 ng/L)	26.6	30.1	27.9	29.1	0.29

BMI: body mass index; 25(OH)D: 25-hydroxyvitamin D; DBP: vitamin D binding protein.

**Table 2:** Selected demographic and study variables of the study sample across frailty categories.

Characteristics	Nonfrail (*N* = 160)	Prefrail (*N* = 182)	Frail (*N* = 174)	P*
Age (y), mean (SD)	72.7 (4.1)	74.6 (5.2)	81.9 (4.4)	<0.05
BMI (kg/m^2^), mean (SD)	26.5 (3.6)	24.3 (5.2)	27.8 (6.1)	0.68
%BMI^†^				0.27
<20.0	4.6	10.3	18.1	
20.0–<25.0	40.2	25.8	26.0	
25.0–<28.0	33.9	29.1	25.6	
≥28.0	21.3	34.8	30.3	
Education (y), mean (SD)	7.8 (2.1)	5.8 (1.6)	4.1 (1.7)	<0.05
Smoking status (% current or previous smokers)	41.2	47.1	45.8	0.62
25(OH)D (nmol/L), mean (SD)	46.1 (11.2)	42.9 (8.3)	35.8 (10.1)	<0.05
DBP (nmol/L), mean (SD)	4576 (1676)	4876 (1319)	5323 (1213)	<0.05
Free 25(OH)D (pmol/L), mean (SD)	26.3 (7.2)	23.4 (5.2)	20.1 (4.3)	0.26
Albumin (g/L), mean (SD)	38.1 (2.1)	37.3 (2.3)	35.7 (2.6)	0.19
Serum calcium (mmol/L), mean (SD)	2.2 (0.2)	2.1 (0.3)	2.0 (0.3)	0.58
Parathyroid hormone (ng/L), mean (SD)	28.9 (7.8)	30.1 (11.6)	32.3 (9.1)	0.62

*Notes*: BMI: body mass index; 25(OH)D: 25-hydroxyvitamin D; DBP: vitamin D binding protein.

*P values were determined using Jonckheere-Terpstra trend test.

^†^BMI was considered a categorical variable as defined and was adjusted in all the regression models.

**Table 3:** Odds ratios of being frail versus nonfrail of participants across quarties of 25(OH)D and DBP levels.

Quarties of 25(OH)D (nmol/L)	Quarties of vitamin D binding protein (nmol/L)			
	Q1b (<4046)	Q2b (4046–<5269)	Q3b (5269–<6686)	Q4b (≥6686)
Q1a (<31.5)	2.63^†^	1.89	1.73	3.18*
Q2a (31.5.3–<41.8)	2.52*	1.95	2.17	2.02
Q3a (41.8–<56.6)	1.36	1.76	1.71	1.82
Q4a (≥56.6)	1.0 (reference)	1.22	1.29	1.37

*Notes*: 25(OH)D: 25-hydroxyvitamin D; DBP: vitamin D binding protein.

*P < 0.05.

^†^ *P* < 0.01.
